# C5a in the peripheral plasma of female fibromyalgia patients is elevated but not related to pain sensitivity as in healthy controls

**DOI:** 10.1038/s41598-025-01347-x

**Published:** 2025-05-19

**Authors:** Koji Fujimoto, Kozo Anno, Yu Tanaka, Masafumi Murakami, Shogo Inamine, Takahiro A. Kato, Nobuyuki Sudo, Masako Hosoi

**Affiliations:** 1 Department of Psychosomatic Medicine, Kyushu University Hospital, Fukuoka, 812-8582 Japan; 2 Multidisciplinary Pain Center, Kyushu University Hospital, Fukuoka, 812-8582 Japan; 3 Department of Psychosomatic Medicine, Fukuoka Dental College Hospital, Fukuoka, 814-0193 Japan; 4 Department of Psychosomatic Medicine, Graduate School of Medical Sciences, Kyushu University, Fukuoka, 812-8582 Japan; 5 Department of Neuropsychiatry, Graduate School of Medical Sciences, Kyushu University, Fukuoka, 812-8582 Japan; 6 Department of Psychiatry, Hokkaido University Graduate School of Medicine, Sapporo, 060-8648 Japan

**Keywords:** Biochemistry, Immunology, Physiology, Biomarkers, Diseases, Medical research

## Abstract

Although evidence from basic studies indicates that C5a induces hyperalgesia, knowledge from studies of humans is limited. This comparative analysis of the peripheral blood C5a concentration of women diagnosed with fibromyalgia (FM) who exhibited widespread pain and that of female healthy controls (HCs) was done to assess possible correlations of C5a concentration with pain threshold. The data of 30 patients with FM and 29 HCs were included in the analysis. C5a concentration in the peripheral blood was quantified by ELISA, and the cold pain threshold (CPT) was assessed. The correlation between C5a concentration and CPT was analyzed using the Spearman correlation coefficient, and the peripheral blood C5a concentrations of FM and HC were compared by t-test. The mean (standard deviation) peripheral blood C5a concentrations of FM and HC were 12.7 (6.48) ng/ml and 8.82 (4.79) ng/ml, respectively (*p* = 0.0114). Although no significant relation was observed between CPT and C5a concentration in FM (R = − 0.12, *p* = 0.52), a significant correlation was found for HC (R = 0.41, p = 0.03). The results suggest that C5a would be a potential biomarker for the pain sensitivity of women and give new insights into the pathophysiology of FM.

## Introduction

Pain is a subjective experience defined as an “unpleasant sensory and emotional experience associated with or resembling that associated with actual or potential tissue damage”^1^. It is estimated that 20.4% of American adults have chronic pain^2^, and the prevalence was estimated at 43.5% in a meta-analysis done in the United Kingdom^3^. The symptoms of pain are not problematic in themselves, but they have a significant negative impact on quality of life^4^ and have been associated with a patient’s social environment, such as when they are in difficult economic circumstances, including homelessness^5^ and divorce^6^.

Fibromyalgia (FM) is a disorder characterized by the presence of widespread severe pain that persists for years. The prevalence was estimated at 1.78% in a population study, with the majority of those affected being women^7^. FM frequently presents with a range of symptoms in addition to pain, including difficulties with morning stiffness, fatigue, non-restorative sleep, concentration, and memory^8^. The precise etiology of FM remains uncertain, though a complex interplay of factors is thought to be involved. In the central nervous system, there are known functional abnormalities in brain regions associated with pain response and processing^9^ as well as psychiatric symptoms such as depression^10,11^. Recently, the Fibromyalgia: Imbalance of Threat and Soothing Systems (FITSS) model has been proposed as a pathophysiologic complex of neural networks and specific cognitive styles for pain^12^. Other pathophysiological concepts include central sensitization, which is defined as an increased pain sensitivity resulting from an increased signaling of pain in the nervous system^13^. It has been suggested that inflammation plays a role in the development of central sensitization. Indeed, mutations in the P2RX7 gene, which is associated with both inflammation and pain, have been linked to an increased risk of developing FM^14^. Moreover, in addition to its relation to the central nervous system, a number of inflammatory mediators have been identified as potential contributors to the development of FM as a systemic abnormality^15^. In a study of patients with FM, 21 of 75 proteins in peripheral blood associated with inflammation were found to be altered in comparison to healthy controls (HCs)^16^.

C5a, a powerful anaphylactic peptide, is generated through the cleavage of C5 into C5a and C5b and plays a pivotal role in all three major pathways of the complement system^17^. C5a administration has been shown to elicit hyperalgesia to both cold and hot stimulus at the site of administration in rodents, and hyperalgesia can be ameliorated by C5a inhibition, indicating that C5a plays a role in its pathogenesis^18–20^. This effect is mediated by the activation of TRPV1 via C5a receptors on macrophages in heat hyperagesia^19^. Paclitaxel produces neuropathic pain, and C5a receptors have been identified as a key mediator in this form of pain, including cold and mechanical allodynia^21,22^. A study that compared the peripheral blood C5a level of patients with neuromyelitis optica (NMO) in remission who were with or without pain found an elevated C5a level in those with pain^23^. Although C5a has been the subject of considerable attention in the context of pain, to the best of our knowledge, no studies have been done of the relation between the C5a level and the pain sensitivity of patients with FM.

Pain is a subjective experience that is challenging to quantify. It is typically evaluated through questionnaires or other subject-reported assessment methods^24^. Questionnaire-based methods frequently employ a visual analog scale (VAS) that represents pain intensity on a 100-mm scale^25^. The numerical Rating scale (NRS) employs either an 11-point or 101-point scale^26^. Because these methodologies rely on patient self-reports, they inherently introduce subjectivity. Due to the intrinsic nature of pain, objective assessment is inherently challenging^27^, underscoring the importance of multidimensional assessment. One relatively simple method of multidimensional pain assessment is the quantitative sensory testing (QST) method^24^, which evaluates the intensity of a potentially painful stimulus when it is actually produced. While the QST does not evaluate the intensity of the pain itself, it can evaluate pain sensitivity and has been standardized^28^. To date, no studies have used it to elucidate the relation between pain sensitivity and peripheral blood C5a.

The objective of the present study was to investigate the following hypotheses: First, that women with FM have higher levels of C5a in their peripheral blood than do healthy women. Second, that the C5a concentration would be related to the pain sensitivity of both groups. We examined the relation between the results of QST and the peripheral blood concentration of C5a in samples of female patients with FM and female HCs.

## Results

**Fig. 1:**
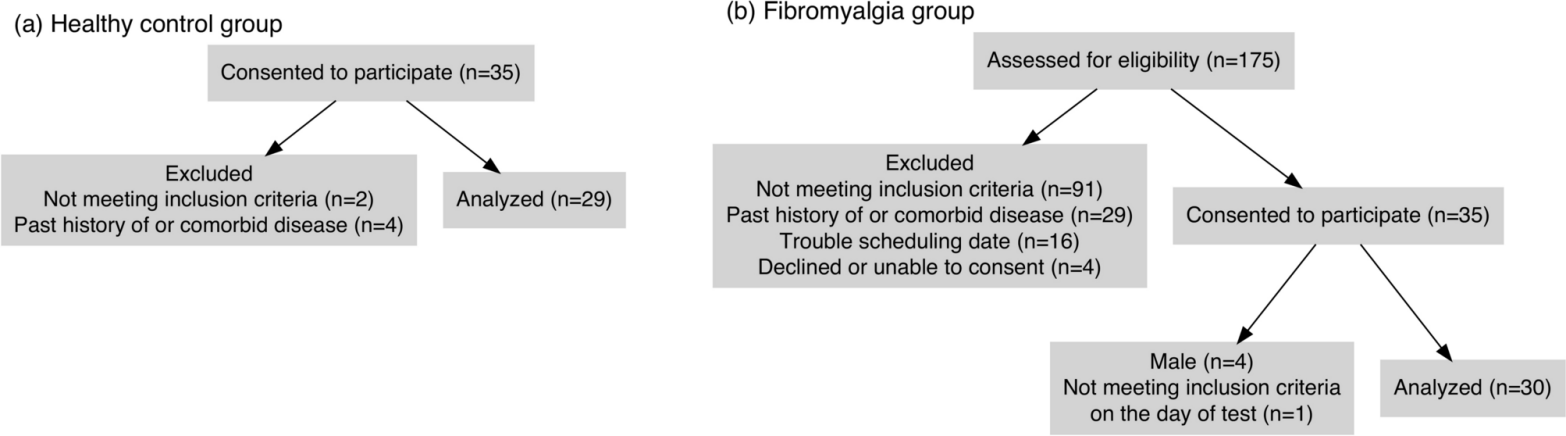
(**a**) Flow diagram of the HC group. 35 women agreed to participate. Exclusions included two participants found to be receiving treatment for pain, one for breast cancer, and three who were suspected of illness on blood screening tests. (**b**) Flow diagram of the FM group. 175 patients were assessed, and 91 patients did not meet the FM criteria. We also excluded 29 patients with diseases that met our exclusion criteria. 16 patients met our inclusion criteria but were not recruited because of cancellation or not being able to be scheduled. Consent could not be obtained from three patients because of a language problem or suspected dementia, one declined to participate. 35 patients agreed to participate, 4 male patients were excluded because of the small number, and one patient had met the inclusion criteria on the day of screening but did not report widespread pain on the testing day.

The data of 30 female patients with FM and 29 female HCs was available for the final analysis (Fig. 1a,b). Consent to participate was obtained from an original cohort of 35 patients and 35 pain-free women recruited as HCs: There were only four male patients, too few to allow for analysis, so they were not included in the study. One patient was excluded who met the inclusion criteria on the day of screening but did not report widespread pain on the testing day. Three HCs did not meet the inclusion criteria, and three more were excluded because they were suspected of having a comorbid illness on screening by blood tests, as delineated in the method section. None of the participants in the HC group had had pain lasting more than 24 hours in the past three months.

The background of the participants of both groups is shown in Table 1. All were women and the age distribution was similar (mean ± SD = 49.97 ± 13.00 (HC)/46.6 ± 13.75 (FM) years, *p* = 0.338). Clinical features are shown in Table 2. The patients had higher pain intensity than HC on VAS (mean ± SD = 5.38 ± 7.27 (HC)/68.51 ± 22.50 (FM) mm, *p* < 0.0001). The cold pain threshold (CPT) of the FM group was significantly higher (more sensitive) than that of the HC group (mean ± SD = 14.52 ± 6.79 (HC)/19.28 ± 7.21 (FM) °C, *p* = 0.013). No difference was observed in the warm pain threshold (WPT, mean ± SD = 42.19 ± 3.72 (HC)/42.7 ± 4.62 (FM) °C, *p* = 0.689): 2 CPTs and 9 WPTs were out of the acceptable range and were treated as missing data. White blood cell count (WBC) was higher in the FM group (mean ± SD = 4.94 ± 1.09 (HC)/6.01 ± 1.78 (FM) ×10^3^/µL, *p* = 0.0076), and the levels of other inflammatory proteins (C-reactive protein) and complement proteins C3 and C4 were not significant.

**Table 1:** Participant background.

		HC (n = 29)	FM (n = 30)	*p*
		Mean ± SD (frequency)	Mean ± SD (frequency)	
Age (years)		49.97 ± 13.00	46.60 ± 13.75	0.338
Martial status	Single	5	9	0.001
	Married	23	10	
	Divorced	0	10	
	Widowed	1	1	
Education (years)	12 < =	6	13	0.114
	12 >	23	17	
Employment	Full-time	3	7	0.013
	Part-time	15	8	
	Homemaker	9	6	
	Student	2	1	
	Unemployed	0	8	

Data collected from self-reported questionnaires. *p* values obtained from t-test for age and chi-squared test for others. Unemployed participants did not include retired. *HC* Healthy control, *FM* Fibromyalgia, *SD* standard deviation.

**Table2:** Clinical features.

	HC (n = 29)		FM (n = 30)		*p*
	n	Mean ± SD	n	Mean ± SD	
VAS (mm)	29	5.38 ± 7.27	30	68.51 ± 22.50	< 0.0001
CPT (°C)	28	14.52 ± 6.79	29	19.28 ± 7.21	0.013
WPT (°C)	23	42.19 ± 3.72	28	42.67 ± 4.62	0.689
WBC (10^3^/µL)	29	4.94 ± 1.09	30	6.01 ± 1.78	0.007
CRP (mg/mL)	29	0.061 ± 0.085	30	0.11 ± 0.181	0.186
C3 (mg/mL)	29	100 ± 16.4	30	105 ± 18.1	0.319
C4 (mg/mL)	29	21.8 ± 6.45	30	23.9 ± 5.86	0.195

2 CPTs and 9 WPTs were out of the measurable range. C3, C4, and CRP were measured by an immunoturbidimetric method, and WBC was measured by flowcytometry. P values were obtained from t-test. *HC* Healthy control, *FM* Fibromyalgia, *SD* Standard deviation, *VAS* Visual analog scale, average pain intensity over 1 week: range from 0 to 100 mm, *CPT* Cold pain threshold, *WPT* Warm pain threshold, *WBC* White blood cell count, *CRP* C-reactive protein.

**Fig. 2:**
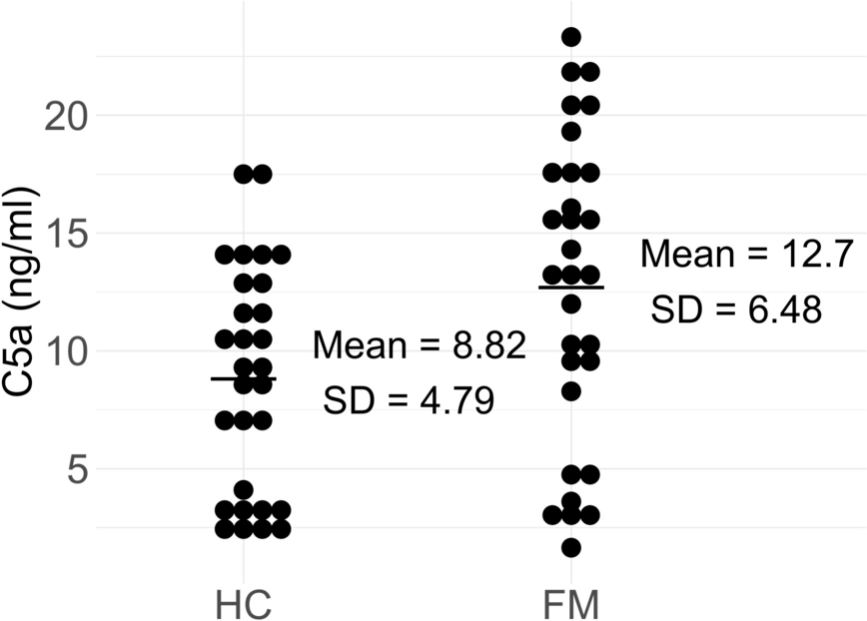
Mean peripheral C5a level in plasma. Means of C5a levels derived from 29 HCs and 30 FMs are compared (*p* = 0.011.t-test), *HC* healthy control, *FM* Fibromyalgia, *SD* standard deviation.

The mean C5a level of the FM group was significantly higher than that of the HC group (Fig. 2, mean ± SD = 8.82 ± 4.79 (HC)/12.7 ± 6.48 (FM) ng/ml, *p* = 0.011). We also measured the levels of other complement proteins with physiological activity (C3a and soluble C5b-9 complex), but the results have been excluded because we were unable to confirm the assay-to-assay reliability of our measurement. No correlation between C5a and its upstream complementary proteins (C3 and C4) was found.

**Fig. 3:**
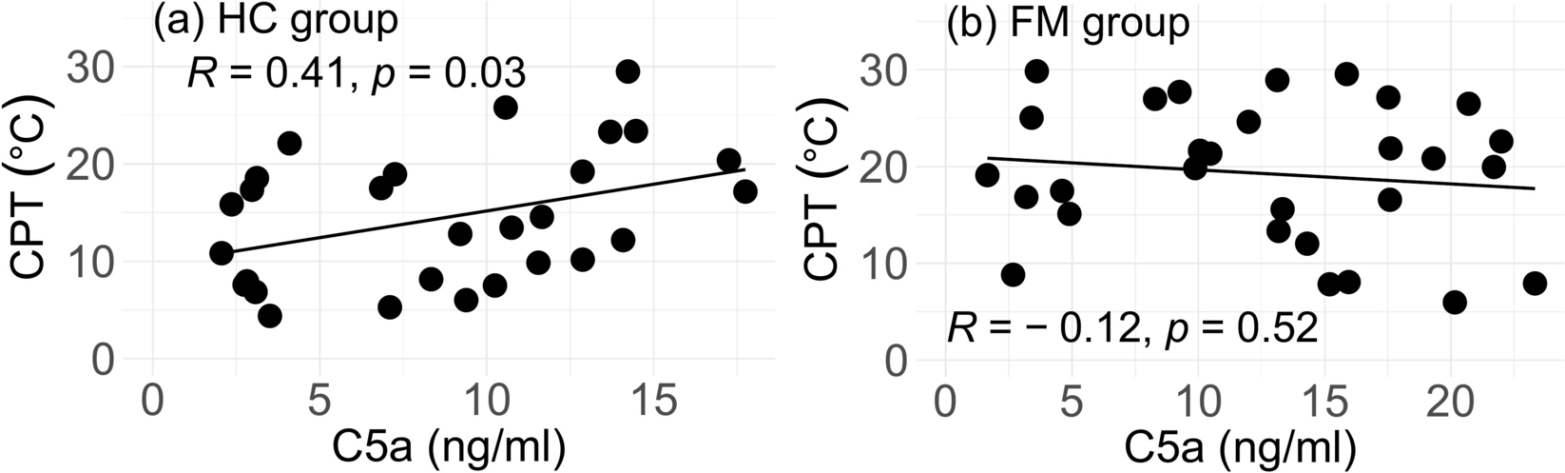
Scatter plot of the C5a and CPT of the HC group (**a**) and FM group (**b**). CPTs of 1 HC and 1 FM were out of the measurable range and omitted. *CPT* Cold pain threshold. P values are obtained from Spearman’s rank correlation. *HC* Healthy control, *FM* Fibromyalgia, *R* Spearman’s rank correlation coefficient.

The clinical features showed a significant, positive correlation between C5a and CPT in the HC group (*R* = 0.41, *p* = 0.03), but no correlation in the FM group (*R* = − 0.12, *p* = 0.52) (Fig. 3a,b). Similarly, a significant, negative correlation was found between C5a and WPT only in the HC group (HC: *R* = − 0.42, *p* = 0.048/FM: *R* = 0.034, *p* = 0.86). No correlation was found between the VAS and C5a in either group (HC: *R* = − 0.064, *p* = 0.74/FM: *R* = − 0.12, *p* = 0.51).

Propensity scoring matched 18 participants from each group. Standardized mean differences (SMD) were evaluated for all covariates, and all of them were below 0.1 after matching. (Supplementary Table 1) The C5a level remained significantly higher in the FM group after matching (mean ± SD = 7.82 ± 4.94 (HC)/12.31 ± 6.96 (FM) ng/ml, *p* = 0.033).

## Discussion

The concentration of C5a in the peripheral blood of the patients with FM in this study was higher than that of HCs, and the C5a concentration and pain threshold of the HC group were negatively correlated. Contrary to our expectation, no correlation was found between C5a concentration and the pain threshold of the FM patients, and there was no correlation between C5a and VAS. The fact that C5a was elevated in these female patients but associated with the pain sensitivity only of the HCs indicates that the way that or degree to which C5a affects pain may be different in healthy women and female FM patients.

A substantial body of research with animal models has investigated the effects of C5a in the context of peripheral pain mechanisms^18–22^. The results of our study are consistent with this, showing that the higher the concentration of C5a in the peripheral blood of these healthy women the more pain they felt. It is notable that the C5a level of the patients with FM of our study was elevated but not related to pain sensitivity. C5a is produced upon activation of the complement pathway and represents a terminal pathway of the complement system. Elevated levels of C5a have been observed in various situations, including those caused by the SARS-CoV-2 virus^29^, antineutrophil cytoplasmic antibody (ANCA) vasculitis^30^, pregnancy^31^, hemolytic uremic syndrome (HUS)^32^, cerebrovascular disease^33^, and exercise^34^. Except for cerebrovascular disease and exercise, these conditions are associated with inflammation, such as from infections and autoimmune diseases. Another possible mechanism is described in a report showing that immunotherapy improved the pain of patients with neuropathic pain who were positive for anti-plexin D1 antibodies^35^. As with patients with neuropathic pain, the possibility of symptom-related autoantibodies in patients with fibromyalgia has been suggested^36^, and Seefried et al.^37^ have reported that the presence of autoantibodies is associated with symptom severity. In such cases, elevated C5a may not be a direct cause of the pain of FM patients and therefore may not be associated with pain sensitivity. In the present study, although no significant difference other than WBC was observed in the background inflammatory response between the patient and control groups, the mean C5a level was elevated in the patient group. This finding suggests that the fibromyalgia cohort is not a homogeneous population, but rather a subset of patients with immune abnormalities, as described above, which may have contributed to the elevated mean C5a level.

In addition to its involvement in peripheral mechanisms, C4, which also belongs to the complement system, has been identified as a contributing factor to schizophrenia that is characterized by symptoms derived from the central nervous system (CNS)^38^. The mechanism of C4-induced schizophrenia is thought to involve synaptic pruning by microglia^39,40^, immune cells that are resident in the CNS. The Goker group suggested a potential association between the C4 level in peripheral blood and clinical manifestations^41^. Microglia are known to express C5a receptors^18^. In a previous study, we reported that microglial dysfunction plays a role in the pathogenesis of FM^42^. Microglial-mediation may be a factor in the relation between FM and C5a, as it is between schizophrenia and C4 in the CNS. In the context of FM, the correlation between C5a and pain threshold to cold stimulus in QST may be obscured by the confluence of microglial-mediated alterations in the pain threshold and abnormalities in brain network function^12^.

WBC and CRP are influenced by inflammation, and C3 and C4 are upstream complementary proteins to C5a. No significant difference in CRP, C3, or C4 was observed at entry in either the FM patients or HCs. However, a significant difference in C5a was found, as was a significant relation in the pain threshold testing. While WBC was higher in patients with FM at entry, C5a remained higher in the FM group after controlling for the WBC values of both groups in the propensity score matching. These findings suggest that C5a has the potential to serve as a useful indicator of FM.

The difference in the mean CPT of FM and HC was significant in this study, while WPT was not, results similar to those of previous studies^43,44^. With CPT used as a QST parameter, a correlation was observed between CPT and the C5a of our HCs, but no such correlation was evident for the VAS of pain. The discrepancy between the QST and self-reported pain score of the healthy participants may be attributed to bias from the distribution of participants with a pain VAS near 0.

Medication may have influenced our findings. Because C5a is an inflammatory protein, it is susceptible to the impact of immunosuppressive medications, such as steroids and biological agents employed in the treatment of collagen diseases. Potential participants who had been prescribed or were currently undergoing treatment with these medications for a designated duration were excluded from the analysis to maintain the integrity of the study. QST results can be also influenced by analgesics and related medications^45,46^. In the analysis of healthy participants, the data of those who had taken these drugs within the previous three months were excluded from the study. In the case of FM patients, only a few had no history of taking these medications. Due to ethical considerations, these medications could not be discontinued, even on the day of testing. The limited sample size of this study precluded sufficient control over the type and amounts of oral medications, underscoring the need for further study in future investigations.

Only female subjects were enrolled in the present study because there were too few men to give an adequate sample size. Regarding C5a and sex, studies using mouse models of disease showed that response to interventions targeting the C5a receptor (C5aR) differed greatly by sex, depending on the disease. For example, in a mouse model of hypoxic encephalopathy, treatment with a C5aR antagonist had a stronger therapeutic effect on female mice^47^, while in an animal study on food allergy, the effect of C5aR deficiency was stronger in males^48^. These findings suggest sex related differences in peripheral blood C5a levels and in the relation between C5a and the pain threshold of FM patients. In the context of QST, women have been reported to be more sensitive to pain and the difference from men has been shown to decrease with age^49,50^. Future studies are needed to examine potential differences between men and women.

Soluble C5b-9 and C3a were not analyzed in this study. Soluble C5b-9 has been identified as a marker that, in conjunction with C5a, reflects the activation of the terminal pathway, a part of the complement system. By observing the activation of soluble C5b-9, it is possible to estimate whether the increase in C5a is due to an increase in C5a alone or whether the entire terminal pathway is activated. Given that the C3a level is indicative of both the activation of the alternative pathway and the overall complement pathway, the elevation of C3a can be utilized to estimate the involvement of the alternative pathway in C5a elevation. Further elucidation of these findings awaits the measurement of these proteins in subsequent studies.

Several C5a antagonists, including orally administered agents, have been developed and are currently in clinical use^51^. In this study, we present, for the first time, evidence of an potential association between C5a and FM as well as a quantitatively derived relation between pain and C5a in healthy women. If C5a is proven to be causally related to pain symptoms, including FM, C5a antagonists would offer a promising new approach to pain management. Further studies will be necessary to confirm this hypothesis.

The following limitations are inherent to this study. First, it should be noted that the assay used measures the inactive form of C5a after it has undergone metabolic transformation. Consequently, caution should be exercised in interpreting the results about the C5a level. C5a is generated by the cleavage of complement component C5 into C5a and C5b. Although C5a is initially biologically active, it is rapidly inactivated through the removal of its C-terminal arginine residue by plasma peptidases, resulting in a less active form of C5a^52^. In this study, the measured C5a represents this inactivated form; therefore, the assay reflects the combined concentration of C5a that was inactivated during collection and measurement and C5a that may have already existed in its inactive form. This methodological limitation implies a potential discrepancy between the physiological concentration of inactive C5a in circulation and the levels detected in our assay. Second, because this is a cross-sectional study, no direct causal relation can be implied. Further longitudinal or interventional investigation will be necessary to determine if C5a causes pain, lowers the pain threshold, or raises it because of other factors. Third, the number of male patients with FM has been shown to be very small compared to female patients in past studies. This was also true in the population recruited for this study. Given the exclusion of male FM patients and the exclusive focus on women, our results cannot be generalized to men. Future research will be needed that includes a sufficient number of men to create an adequate sample size. Fourth, despite the fact that complications and comorbidities were meticulously recorded by interview and referrals were validated, it is plausible that undiagnosed or unexplained comorbidities may exist. Fifth, the limited sample size posed significant challenges to effectively controlling for possible confounding effects of medication use. Sixth, the limited sample size engenders an elevated risk of false-positive findings, which may arise from inherent inaccuracies or biases within the measurement system. To avoid this problem, we carefully followed the manufacturer’s instructions for the measurements of complemental proteins and performed the measurements several times with the same methodology (environment and measuring kit from the same manufacturer). Future study with larger samples and the use of different assays will be needed to confirm the results of this study. Seventh, missing values exist for the QST results, but again, due to sample size issues, we were not able to conduct a sensitivity analysis or multiple imputation that excluded these values. This needs to be considered in future research. Eighth, recruitment for this study spanned a period of over a year, the QST and blood samples were measured at a constant room temperature (22–24 °C), the QST measurements were initiated after the skin temperature had reached a consistent level, and blood samples were kept on ice or 4 °C until storing at − 80 °C or measurement; however, it is possible that external temperature fluctuations may have influenced the results.

In conclusion, our results indicate that C5a has potential as a biomarker for the pain of healthy women and provide new insights into the pathogenesis of FM. Longitudinal studies large enough to include male patients are needed to further clarify the role of C5a in FM.

## Methods

### Study design

This is a cross-sectional study aimed at comparing the level of C5a in the peripheral plasma of patients who met the criteria for FM and a group of HCs to determine the relation between C5a concentration and clinical features, especially pain.

### Ethical considerations and study registration

This study was conducted at the department of psychosomatic medicine, Kyushu University Hospital, with approval from the Kyushu University Institutional Review Board for Clinical Research (#22096-1). All participants provided written informed consent prior to participation. The study was performed in accordance with the Declaration of Helsinki and the laws and regulations of Japan, and it was registered with UMIN-CTR (#UMIN000048852), which ensures transparency and allows for public access to the study protocol and updates.

### Participants

A consecutive series of female FM patients and HCs with similar attributes were recruited for study. The FM group was comprised of outpatients who visited the department of psychosomatic medicine, Kyushu University for the first time with a chief complaint of pain between September 12th, 2022 and March 31st, 2024. HCs were recruited from a community mailing list. Inclusion criteria were as follows: FM group, patients who met the American college of rheumatology preliminary diagnostic criteria for FM^53,54^. HC group, women who had not experienced pain lasting more than 24 h in the past three months. All participants were 18 years or older, had the ability to provide informed consent, and showed a willingness to participate in the study. Exclusion criteria included any unsuitable conditions such as those identified by blood tests, anemia, or any chronic illness that might cause pain: autoimmune diseases, chronic infection, demyelinating disease, complement-related diseases, and malignant diseases: which was confirmed by collecting information from the participants themselves and by the patient referral documents sent to us by the patient’s clinician. All persons considered for the HC group who were receiving any treatment for pain were excluded. All participants in the HC group underwent blood tests for screening. All participants received a compensation of 2000 yen to acknowledge the time and effort involved in completing the study procedures.

### Sample size

A continuous response variable from the independent controls and experimental subjects was used, with one control per experimental subject. Using the data of a previous study^33^ in which the response within each subject group was normally distributed, with a difference in the means of 10.1 and a standard deviation of 11.1, we would need to study 20 experimental subjects and 20 controls to be able to reject the null hypothesis that the population means of the experimental and control groups are equal with a probability (power) of 0.8. The Type I error probability associated with this test of this null hypothesis is 0.05.

### Data collection

Blood samples were collected from all eligible participants. Peripheral venous blood samples were drawn into EDTA tubes, stored on ice until centrifugation, and EDTA plasma was separated by centrifugation at 1200 × g for 15 min at 4 °C. Centrifugation was done no longer than two hours after blood was drawn. The plasma samples were then aliquoted and stored at – 80 °C until analysis. The participants finished their physical examination on the same day as blood collection, and questionnaires were filled out that day or within the prior week.

### Measurement of complement and inflammatory markers

The plasma C5a level was measured by the use of MicroVue Complement C5a EIA (QuidelOrtho, USA), C3a with MicroVue Complement C3a Plus EIA (QuidelOrtho, USA), and Soluble C5b-9 with MicroVue complement SC5b-9 Plus EIA (QuidelOrtho, USA). All measurements were made according to the manufacturers’ instructions. The assays used were those available in Japan that had a stable supply. C3, C4, and CRP were measured by an immunoturbidimetric method, and WBC was measured by flowcytometry.

### Participant background

The collection of clinical information was by questionnaire. The information collected included sex, age, marital status (never married, married, divorced, or widowed), education (years), employment (full time, part time, temporally unemployed, student, or stay-at-home parent), and average pain intensity over a week on a VAS of 0 to 100 mm.

### Quantitative sensory testing (QST)

Pain sensitivity was measured by QST, as in a previous study^28^. Intercross-220 (Intercross corporation, Japan) was used to assess CPT and WPT. It was obtained with ramped stimuli (1 °C/s) from a baseline temperature (32 ± 0.5 °C) and was terminated when the participant felt pain and pressed a button. The mean threshold temperature was calculated from three consecutive measurements. The contact area was 26.22 cm^2^. Because of the risk of burns and frostbite, the range of temperature change was 0–50 °C. If the patient did not report pain within this range, the data was treated as a missing value.

### Statistical analysis

The independent samples t-test was used for comparisons of the concentration of each complement. Correlation between the level of a complement and clinical features (CPT and VAS) was by Spearman’s rank correlation coefficient. A *p*-value < 0.05 was considered statistically significant. Analyses were performed with R software, version 4.4.1.

### Sensitivity analysis

Because complements are inflammatory mediators, we used propensity score matching to control the background inflammatory levels and compared the concentration of complements by the independent samples t-test. The propensity score was estimated with a logistic regression model using the following covariates: age, WBC, and the CRP, C3, and C4 levels. The neighbor matching method was used, with a caliper value of 0.17. SMD < 0.1 was considered acceptable.

## Supplementary Information

Supplementary Information.

## Data Availability

The anonymized datasets generated during the current study are available from the corresponding author on reasonable request.

## References

[1] Raja, S. N. et al. The revised international association for the study of pain definition of pain: concepts, challenges, and compromises. *Pain* **161**, 1976–1982 (2020). 32694387 10.1097/j.pain.0000000000001939 PMC7680716

[2] Dahlhamer, J. et al. Prevalence of chronic pain and high-impact chronic pain among adults—United States, 2016. *MMWR. Morb. Mortal. Wkly. Rep.* **67**, 1001–1006 (2018). 30212442 10.15585/mmwr.mm6736a2 PMC6146950

[3] Fayaz, A., Croft, P., Langford, R. M., Donaldson, L. J. & Jones, G. T. Prevalence of chronic pain in the UK: A systematic review and meta-analysis of population studies. *BMJ Open* **6**, e010364 (2016). 27324708 10.1136/bmjopen-2015-010364 PMC4932255

[4] Clauw, D. J., Essex, M. N., Pitman, V. & Jones, K. D. Reframing chronic pain as a disease, not a symptom: rationale and implications for pain management. *Postgrad. Med.* **131**, 185–198 (2019). 30700198 10.1080/00325481.2019.1574403

[5] Fisher, R. et al. The nature and prevalence of chronic pain in homeless persons: an observational study. *F1000Research* **2**, 164 (2013). 24555079 10.12688/f1000research.2-164.v1 PMC3886796

[6] de Vieira, É. B. M. et al. Chronic pain, associated factors, and impact on daily life: are there differences between the sexes?. *Cad. Saude Publica* **28**, 1459–1467 (2012). 22892966 10.1590/s0102-311x2012000800005

[7] Heidari, F., Afshari, M. & Moosazadeh, M. Prevalence of fibromyalgia in general population and patients, a systematic review and meta-analysis. *Rheumatol. Int.* **37**, 1527–1539 (2017). 28447207 10.1007/s00296-017-3725-2

[8] Bennett, R. M., Jones, J., Turk, D. C., Russell, I. J. & Matallana, L. An internet survey of 2596 people with fibromyalgia. *BMC Musculoskelet. Disord.* **8**, 27 (2007). 17349056 10.1186/1471-2474-8-27 PMC1829161

[9] Liu, A. et al. Altered whole brain functional activity in patients with fibromyalgia. *Clin. Exp. Rheumatol.* **42**, 1164–1169 (2024). 38294039 10.55563/clinexprheumatol/ntlvv6

[10] Clauw, D. J. Fibromyalgia: A clinical review. *JAMA* **311**, 1547–1555 (2014). 24737367 10.1001/jama.2014.3266

[11] Gracely, R. H. et al. Pain catastrophizing and neural responses to pain among persons with fibromyalgia. *Brain* **127**, 835–845 (2004). 14960499 10.1093/brain/awh098

[12] Pinto, A. M. et al. Emotion regulation and the salience network: a hypothetical integrative model of fibromyalgia. *Nat. Rev. Rheumatol.* **19**, 44–60 (2023). 36471023 10.1038/s41584-022-00873-6

[13] Yunus, M. B. Central sensitivity syndromes: A new paradigm and group nosology for fibromyalgia and overlapping conditions, and the related issue of disease versus illness. *Semin. Arthritis Rheum.* **37**, 339–352 (2008). 18191990 10.1016/j.semarthrit.2007.09.003

[14] Zorina-Lichtenwalter, K. et al. Characterization of common genetic variants in P2RX7 and their contribution to chronic pain conditions. *J. Pain* **25**, 545–556 (2024). 37742908 10.1016/j.jpain.2023.09.011

[15] Ji, R.-R., Nackley, A., Huh, Y., Terrando, N. & Maixner, W. Neuroinflammation and central sensitization in chronic and widespread pain. *Anesthesiology* **129**, 343–366 (2018). 29462012 10.1097/ALN.0000000000002130 PMC6051899

[16] Bäckryd, E., Tanum, L., Lind, A., Larsson, A. & Gordh, T. Evidence of both systemic inflammation and neuroinflammation in fibromyalgia patients, as assessed by a multiplex protein panel applied to the cerebrospinal fluid and to plasma. *J. Pain Res.* **10**, 515–525 (2017). 28424559 10.2147/JPR.S128508 PMC5344444

[17] Zipfel, P. F. & Skerka, C. Complement regulators and inhibitory proteins. *Nat. Rev. Immunol.* **9**, 729–740 (2009). 19730437 10.1038/nri2620

[18] Griffin, R. S. et al. Complement induction in spinal cord microglia results in anaphylatoxin C5a-mediated pain hypersensitivity. *J. Neurosci.* **27**, 8699–8708 (2007). 17687047 10.1523/JNEUROSCI.2018-07.2007 PMC6672952

[19] Shutov, L. P. et al. The complement system component C5a produces thermal hyperalgesia via macrophage-to-nociceptor signaling that requires NGF and TRPV1. *J. Neurosci.* **36**, 5055–5070 (2016). 27147658 10.1523/JNEUROSCI.3249-15.2016 PMC4854968

[20] Warwick, C. A., Shutov, L. P., Shepherd, A. J., Mohapatra, D. P. & Usachev, Y. M. Mechanisms underlying mechanical sensitization induced by complement C5a: the roles of macrophages, TRPV1 and CGRP receptors. *Pain* **160**, 702–711 (2019). 30507785 10.1097/j.pain.0000000000001449 PMC6377341

[21] Cristiano, C. et al. Inhibition of C5aR1 as a promising approach to treat taxane-induced neuropathy. *Cytokine* **171**, 156370 (2023). 37722320 10.1016/j.cyto.2023.156370

[22] Brandolini, L. et al. Paclitaxel binds and activates C5aR1: A new potential therapeutic target for the prevention of chemotherapy-induced peripheral neuropathy and hypersensitivity reactions. *Cell Death Dis.* **13**, 500 (2022). 35614037 10.1038/s41419-022-04964-w PMC9130998

[23] Tong, Y. *et al.* Association of pain with plasma C5a in patients with neuromyelitis optica spectrum disorders during remission. *Neuropsychiatr. Dis. Treat.* **18**, 1039–1046 (2022). 10.2147/NDT.S359620 PMC9124695 35615424

[24] Fillingim, R. B., Loeser, J. D., Baron, R. & Edwards, R. R. Assessment of chronic pain: Domains, methods, and mechanisms. *J. Pain* **17**, T10–T20 (2016). 27586827 10.1016/j.jpain.2015.08.010 PMC5010652

[25] Dworkin, R. H. et al. Core outcome measures for chronic pain clinical trials: IMMPACT recommendations. *Pain* **113**, 9–19 (2005). 15621359 10.1016/j.pain.2004.09.012

[26] McGrath, P. J. et al. Core outcome domains and measures for pediatric acute and chronic/recurrent pain clinical trials: PedIMMPACT recommendations. *J. Pain* **9**, 771–783 (2008). 18562251 10.1016/j.jpain.2008.04.007

[27] Breivik, H. et al. Assessment of pain. *Br. J. Anaesth.* **101**, 17–24 (2008). 18487245 10.1093/bja/aen103

[28] Rolke, R. et al. Quantitative sensory testing in the German Research Network on Neuropathic Pain (DFNS): Standardized protocol and reference values. *Pain* **123**, 231–243 (2006). 16697110 10.1016/j.pain.2006.01.041

[29] Trivedi, V. S. *et al.* Targeting the complement–sphingolipid system in COVID-19 and Gaucher diseases: Evidence for a new treatment strategy. *Int. J. Mol. Sci.* **23**, (2022). 10.3390/ijms232214340 PMC9695449 36430817

[30] Trivioli, G. & Vaglio, A. The rise of complement in ANCA-associated vasculitis: from marginal player to target of modern therapy. *Clin. Exp. Immunol.* **202**, 403–406 (2020). 32946609 10.1111/cei.13515 PMC7670158

[31] Balduit, A. *et al.* Systematic review of the complement components as potential biomarkers of pre-eclampsia: pitfalls and opportunities. *Front. Immunol.* **15**, (2024). 10.3389/fimmu.2024.1419540 PMC11232388 38983853

[32] Raina, R. et al. Systematic review of atypical hemolytic uremic syndrome biomarkers. *Pediatr. Nephrol.* **37**, 1479–1493 (2022). 35118546 10.1007/s00467-022-05451-2

[33] Van Dijk, B. J. et al. Complement C5 contributes to brain injury after subarachnoid hemorrhage. *Transl. Stroke Res.* **11**, 678–688 (2020). 31811640 10.1007/s12975-019-00757-0 PMC7340633

[34] Rothschild-rodriguez, D., Causer, A. J., Brown, F. F., Collier-bain, H. D. & Moore, S. The effects of exercise on complement system proteins in humans: A systematic scoping review The effects of exercise on complement system proteins in humans: A systematic scoping review. *Exerc. Immunol. Rev.* **28**, 1–35 (2022). 35452398

[35] Fujii, T. et al. A novel autoantibody against Plexin D1 in patients with neuropathic pain. *Ann. Neurol.* **84**, 200–224 (2018). 30014510 10.1002/ana.25279

[36] Goebel, A. *et al.* Passive transfer of fibromyalgia pain from patients to mice. *J. Clin. Investig.* **131**, (2019). 10.1172/JCI144201 PMC8245181 34196305

[37] Seefried, S. *et al.* Autoantibodies in patients with fibromyalgia syndrome. *Pain*. 10.1097/j.pain.0000000000003535 (2025) 10.1097/j.pain.0000000000003535 39907533

[38] Sekar, A. et al. Schizophrenia risk from complex variation of complement component 4. *Nature* **530**, 177–183 (2016). 26814963 10.1038/nature16549 PMC4752392

[39] Sellgren, C. M. et al. Increased synapse elimination by microglia in schizophrenia patient-derived models of synaptic pruning. *Nat. Neurosci.* **22**, 374–385 (2019). 30718903 10.1038/s41593-018-0334-7 PMC6410571

[40] Yilmaz, M. *et al.* Overexpression of schizophrenia susceptibility factor human complement C4A promotes excessive synaptic loss and behavioral changes in mice. *Nat. Neurosci.* **24**, (2021). 10.1038/s41593-020-00763-8 PMC8086435 33353966

[41] Goker, M., Aytac, H. M. & Guclu, O. Evaluation of serum complement levels and factors affecting treatment resistance in patients with schizophrenia. *Psychiatory Clin. Psychopharmacol.* **33**, 84–93 (2023). 10.5152/pcp.2023.22580 PMC11082620 38765923

[42] Ohgidani, M. et al. Fibromyalgia and microglial TNF-α: Translational research using human blood induced microglia-like cells. *Sci. Rep.* **7**, 11882 (2017). 28928366 10.1038/s41598-017-11506-4 PMC5605512

[43] Weber, T. et al. Fibromyalgia-associated hyperalgesia is related to psychopathological alterations but not to gut microbiome changes. *PLoS ONE* **17**, e0274026 (2022). 36149895 10.1371/journal.pone.0274026 PMC9506607

[44] Berwick, R. J., Siew, S., Andersson, D. A., Marshall, A. & Goebel, A. A systematic review into the influence of temperature on fibromyalgia pain: meteorological studies and quantitative sensory testing. *J. Pain* **22**, 473–486 (2021). 33421589 10.1016/j.jpain.2020.12.005

[45] Zhang, Y. et al. Increased pain sensitivity in chronic pain subjects on opioid therapy: A cross-sectional study using quantitative sensory testing. *Pain Med. (United States)* **16**, 911–922 (2015). 10.1111/pme.12606 25376890

[46] Olesen, S. S. et al. Quantitative sensory testing predicts pregabalin efficacy in painful chronic pancreatitis. *PLoS ONE* **8**, e57963 (2013). 23469256 10.1371/journal.pone.0057963 PMC3585877

[47] Saadat, A. et al. Structural and functional effects of C5aR1 antagonism in a rat model of neonatal hypoxic-ischemic encephalopathy. *Dev. Neurosci.*10.1159/000539506 (2024). 38797164 10.1159/000539506 PMC11965858

[48] Kordowski, A. et al. C5a receptor 1 −/− mice are protected from the development of IgE-mediated experimental food allergy. *Allergy* **74**, 767–779 (2019). 30341777 10.1111/all.13637

[49] Neziri, A. Y. et al. Reference values of mechanical and thermal pain tests in a pain-free population. *Eur. J. Pain* **15**, 376–383 (2011). 20932788 10.1016/j.ejpain.2010.08.011

[50] Vogel, K. et al. Sex differences in pain and quantitative sensory testing in patients with rheumatoid arthritis. *Arthritis Care Res.* **75**, 2472–2480 (2023). 10.1002/acr.25178 PMC10704379 37365745

[51] Mohebnasab, M. et al. Current and future approaches for monitoring responses to anti-complement therapeutics. *Front. Immunol.* **10**, 2539 (2019). 31787968 10.3389/fimmu.2019.02539 PMC6856077

[52] Hugli, T. E. Structure and function of the anaphylatoxins. *Springer Semin. Immunopathol.* **7**, 193–219 (1984). 6387982 10.1007/BF01893020

[53] Wolfe, F. et al. The American College of Rheumatology preliminary diagnostic criteria for fibromyalgia and measurement of symptom severity. *Arthritis Care Res.* **62**, 600–610 (2010). 10.1002/acr.20140 20461783

[54] Wolfe, F. et al. 2016 Revisions to the 2010/2011 fibromyalgia diagnostic criteria. *Semin. Arthritis Rheum.* **46**, 319–329 (2016). 27916278 10.1016/j.semarthrit.2016.08.012

