# Supplementary material for: C5a in the peripheral plasma of female fibromyalgia patients is elevated but not related to pain sensitivity as in healthy controls
Source: Sci Rep. 2025 May 19;15:17387. doi: 10.1038/s41598-025-01347-x (PMC12089290; doi:10.1038/s41598-025-01347-x)
Supplement: Supplementary file 1 — Supplementary Information. [file 41598_2025_1347_MOESM1_ESM.docx]

|  | **Unmatched HC** | | **Unmatched**  **FM** | | **Unmatched** | **Matched**  **HC** | | **Matched**  **FM** | | **Matched** |
| --- | --- | --- | --- | --- | --- | --- | --- | --- | --- | --- |
|  | **Mean** | **SD** | **Mean** | **SD** | **SMD** | **Mean** | **SD** | **Mean** | **SD** | **SMD** |
| **WBC** | 4.94 | 1.09 | 6.01 | 1.78 | 0.60 | 5.34 | 1.11 | 5.31 | 1.14 | -0.01 |
| **Age** | 50.0 | 13.0 | 46.6 | 13.8 | -0.24 | 48.7 | 14.1 | 49.4 | 13.5 | 0.05 |
| **C3** | 100.6 | 16.4 | 105.1 | 18.1 | 0.25 | 104.6 | 17.2 | 103.3 | 16.4 | -0.07 |
| **C4** | 21.8 | 6.45 | 23.9 | 5.86 | 0.36 | 22.8 | 4.97 | 23.3 | 6.49 | 0.08 |
| **lnCRP** | -3.42 | 1.05 | -2.99 | 1.22 | 0.36 | -3.31 | 1.19 | -3.21 | 0.92 | 0.08 |

**Supplementary table1**. HC: healthy control, FM: fibromyalgia, SD: standard deviation, SMD: standardized mean differences, WBC: white blood cell count, lnCRP: logarithmically transformed C- reactive protein. Units are following: WBC 10^3^/uL, Age year, C3 mg/mL, C4 mg/mL, lnCRP mg/mL.
